# Antibacterial activity of chlorhexidine alone and in combination with metronidazole, *Punica granatum*, and *Curcuma longa* against *Porphyromonas gingivalis*: an in vitro study

**DOI:** 10.2340/aos.v85.46854

**Published:** 2026-09-29

**Authors:** Nujud Alamry, Shatha S. ALHarthi, Shorouq Subhi ALHarthi, Najla Naif Al-Dabbagh, Manal Matouq Shalabi, Shahad Ali Alsaqami, Afrah E. Mohammed, Kawther Aabed

**Affiliations:** a Department of Preventive Dental Sciences, College of Dentistry, Princess Nourah Bint Abdulrahman University, Riyadh, Saudi Arabia; b Department of Organizational Excellence-Quality, Saudi Center Board for Accreditation of Healthcare Institutions, Riyadh, Saudi Arabia; c Periodontics Department, Faculty of Dentistry, King Abdulaziz University, Jeddah, Saudi Arabia; d Dental Intern, College of Dentistry, Princess Nourah bint Abdulrahman University, Riyadh, Saudi Arabia; e Research Department, Natural and Health Sciences Research Center, Princess Nourah Bint Abdulrahman University, Riyadh, Saudi Arabia; f Department of Biology, College of Science, Princess Nourah Bint Abdulrahman University, Riyadh, Saudi Arabia

**Keywords:** Chlorhexidine, metronidazole, *Porphyromonas gingivalis*, periodontitis, antibacterial agents

## Abstract

**Background:**

The aim was to evaluate the antibacterial activity of 0.2% chlorhexidine (CHX) alone and in combination with metronidazole, *Punica granatum* extract, and *Curcuma longa* extract against *Porphyromonas gingivalis*.

**Materials and Methods:**

An in vitro experimental study was performed using *P. gingivalis* strain ATCC 33277. Bacteria were cultured under anaerobic conditions. Ten experimental groups were evaluated: 0.2% CHX, 0.5% metronidazole, 100% *P. granatum* extract, 0.1 and 1.0% *C. longa* extracts, their respective combinations with 0.2% CHX, and distilled water. Antibacterial activity was assessed using an agar-well diffusion assay, and the diameter of the bacterial growth inhibition zone was measured in millimeters. Statistical significance was established at *P* < 0.05.

**Results:**

The combination of 0.2% CHX and 0.5% metronidazole demonstrated the greatest antibacterial activity, producing the largest inhibition zone (21.67 ± 1.60 mm). This value was significantly greater than those observed for CHX alone (16.44 ± 1.09 mm, *P* < 0.01), *P. granatum* combined with CHX (18.11 ± 0.11 mm, *P* < 0.01), 0.1% *C. longa* combined with CHX (18.89 ± 0.40 mm, *P* < 0.01), and 1.0% *C. longa* combined with CHX (18.40 ± 0.62 mm, *P* < 0.01). Among the individual agents, 100% *P. granatum* extract demonstrated the greatest antibacterial activity (20.67 ± 0.00 mm), whereas metronidazole alone, both *C. longa* extracts, and the negative control produced no measurable inhibition zones.

**Conclusions:**

The combination of CHX and metronidazole exhibits the strongest antibacterial activity, whereas *P. granatum* extracts demonstrate substantial antibacterial activity against *P. gingivalis*.

## Introduction

Poor daily oral hygiene maintenance is the classical clinical risk factor for oral diseases, including periodontitis [1, 2]. However, from a microbiological aspect, periodontitis results from dysbiosis in the subgingival biofilm that elicits an exaggerated host inflammatory response, with Gram-negative anaerobic bacteria playing significant roles in disease initiation and progression [3–5]. Among these microorganisms, *Porphyromonas gingivalis* has been labeled as a major pathogen due to its ability to evade host immune defenses, disrupt microbial homeostasis, and promote chronic inflammation [6–8]. Therefore, suppression of *P. gingivalis* remains a major therapeutic objective in periodontal treatment. Mechanical debridement represents the cornerstone of periodontal therapy [9–12]; however, eliminating periodontal pathogens from deep periodontal pockets and complex biofilm structures is often challenging. In this context, residual bacterial colonization may contribute to persistent inflammation and disease recurrence, thereby justifying the adjunctive use of antimicrobial agents [13].

Chlorhexidine (CHX), particularly at concentrations of 0.12–0.2%, remains the gold-standard antiseptic in dentistry owing to its broad-spectrum antimicrobial activity, substantivity, and proven efficacy in reducing dental plaque and gingival inflammation [14, 15]. However, combination antimicrobial therapy has emerged as a promising approach to improve antibacterial efficacy through combined interactions [16–18]. Metronidazole is a nitroimidazole antimicrobial agent that is highly effective against obligate anaerobic bacteria, including *P. gingivalis* [19, 20]. It disrupts bacterial DNA synthesis and has been widely used as both a systemic and locally delivered adjunct in periodontal therapy [19, 20]. An in vitro study [21] showed that CHX and metronidazole exhibit significant antimicrobial activity against *P. gingivalis*, resulting in 50 and 99.9% reductions in bacterial growth, respectively. However, growing concerns regarding antimicrobial resistance and the increasing demand for naturally derived therapeutic agents have stimulated research into plant-based compounds with antimicrobial and anti-inflammatory properties. *Curcuma longa* contains curcumin, a polyphenolic compound exhibiting antimicrobial, antioxidant, and anti-inflammatory activities [22, 23]. Although *C. longa* has demonstrated inhibitory effects against *P. gingivalis* under optimized laboratory conditions, its poor aqueous solubility and limited bioavailability considerably restrict its antimicrobial performance in conventional formulations [24]. Similarly, punicalagin-rich extracts derived from *Punica granatum*, which are abundant in the water-soluble antioxidant polyphenol punicalagin, have demonstrated significant antibacterial and antibiofilm activity against periodontal pathogens [25, 26]. Despite such encouraging results [24–26], comparative evidence evaluating these natural products individually and in combination with CHX against *P. gingivalis* remains limited.

The aim of the present in vitro study is to evaluate the antibacterial activity of CHX alone and in combination with metronidazole, *P. granatum*, and *C. longa* extract against *P. gingivalis*. The null hypothesis is that combining CHX with metronidazole, *P. granatum*, or *C. longa* does not produce greater antibacterial activity against *P. gingivalis* than CHX alone.

## Materials and methods

### Ethical guidelines and study design

This study was exempt from ethical approval and informed consent requirements, as it involved exclusively laboratory-based in vitro experiments using bacterial cultures without the involvement of human participants, human tissues, human-derived biological specimens, and/or experimental animals. Accordingly, institutional ethics committee review was not required under applicable institutional and national research guidelines.

### Bacterial strain and culture conditions

The *P. gingivalis* ATCC 33277 (American Type Culture Collection, Manassas, VA, USA) was used as the test microorganism. The bacteria were cultured on supplemented Brucella blood agar containing 5% defibrinated sheep blood, 5 µg/mL hemin, and 1 µg/mL menadione (vitamin K₁), and incubated at 37°C for 48 h under anaerobic conditions consisting of 80% N₂, 10% H₂, and 10% CO₂. Fresh bacterial colonies were suspended in sterile phosphate-buffered saline (PBS) and adjusted to a turbidity equivalent to a 0.5 McFarland standard (approximately 1.5 × 10⁸ CFU/mL) prior to antimicrobial testing.

### Preparation of antimicrobial agents

Metronidazole and *P. granatum* extract were prepared at concentrations of 0.5 and 100%, respectively. The *C. longa* preparations were evaluated at concentrations of 1 mg/mL (0.1%) and 10 mg/mL (1%). Commercially available 0.2% CHX gluconate solution and sterile distilled water were used as positive and negative controls, respectively. Combination groups consisted of each test material mixed with 0.2% CHX immediately before antimicrobial testing.

### Experimental groups

The antibacterial activity of the test agents was evaluated using the following experimental groups: (1) 0.2% CHX; (2) 0.5% metronidazole; (3) a combination of 0.5% metronidazole and 0.2% CHX; (4) 100% *P. granatum* extract; (5) a combination of 100% *P. granatum* extract and 0.2% CHX; (6) 0.1% *C. longa* extract; (7) 1.0% *C. longa* extract; (8) a combination of 0.1% *C. longa* extract and 0.2% CHX; (9) a combination of 1.0% *C. longa* extract and 0.2% CHX; and (10) sterile distilled water.

### Assessment of antibacterial activity and outcome measure

Antibacterial activity was assessed using the agar-well diffusion method. A standardized suspension of *P. gingivalis* equivalent to a 0.5 McFarland standard was uniformly inoculated onto the surface of supplemented Brucella blood agar plates using a sterile swab. After inoculation, circular wells were aseptically prepared in the agar using a sterile cork borer. An equal volume of each test solution was dispensed into the respective wells. The plates were subsequently incubated at 37°C for 48 h under anaerobic conditions. Following incubation, antibacterial activity was determined by measuring the diameter of the bacterial inhibition zone, in millimeters. All experiments were performed in triplicate.

### Statistical analysis

All experiments were performed in triplicate, and the data are presented as mean ± standard deviation (SD). Statistical analyses were conducted using IBM SPSS Statistics for Windows, version 29.0 (IBM Corp., Armonk, NY, USA). Data normality was assessed using the Shapiro–Wilk test. Differences among the experimental groups were analyzed using one-way analysis of variance (ANOVA), followed by Tukey’s post hoc test for multiple pairwise comparisons. Statistical significance was set at *P* < 0.05.

## Results

To directly address the null hypothesis, pairwise comparisons were performed between 0.2% CHX alone and each CHX-containing combination. Compared with CHX alone (16.44 ± 1.09 mm), the CHX–metronidazole combination produced a significantly greater inhibition zone (21.67 ± 1.60 mm; *P* < 0.01). The CHX–*P. granatum* combination (18.11 ± 0.11 mm), CHX–0.1% *C. longa* combination (18.89 ± 0.40 mm), and CHX–1.0% *C. longa* combination (18.40 ± 0.62 mm) also showed numerically greater inhibition zones than CHX alone; however, the statistical significance of these pairwise comparisons should be reported explicitly. The CHX–metronidazole combination demonstrated the greatest antibacterial activity among all CHX-containing combinations. In the present study, 0.5% metronidazole, 0.1% *C. longa* extract, 1.0% *C. longa* extract, and sterile distilled water produced no measurable inhibition zones under the agar-diffusion assay conditions (Table 1).

**Table 1:** Antibacterial activity of chlorhexidine, metronidazole, pomegranate extract, and *Curcuma longa* extracts against *Porphyromonas gingivalis*.

Experimental group	Inhibition zone (mm), mean ± SD
Sterile distilled water	0.00 ± 0.00
CHX (0.2%)*	16.44 ± 1.09
Metronidazole (0.5%)	0.00 ± 0.00
*C. longa* extract (0.1%)	0.00 ± 0.00
*C. longa* extract (1.0%)	0.00 ± 0.00
*P. granatum* extract (100%)	20.67 ± 0.00
*P. granatum* extract (100%) + Chlorhexidine (0.2%)*	18.11 ± 0.11
*C. longa* extract (0.1%) + Chlorhexidine (0.2%)*	18.89 ± 0.40
*C. longa* extract (1.0%) + Chlorhexidine (0.2%)*	18.40 ± 0.62
CHX (0.2%) + Metronidazole (0.5%)	21.67 ± 1.60

P. granatum: Punica granatum; C. longa: Curcuma longa; SD: standard deviation; CHX: Chlorhexidine.

Values are presented as mean ± standard deviation (mm).

* Compared with Chlorhexidine (0.2%) + Metronidazole (0.5%) (*P* < 0.01).

## Discussion

In summary, the principal finding of this in vitro study was that the combination of 0.2% CHX and 0.5% metronidazole showed the greatest antibacterial activity, producing the largest inhibition zone, which was significantly greater than CHX alone and all other combination therapies. Moreover, *P. granatum* extract alone demonstrated substantial antibacterial activity, whereas metronidazole alone and both *C. longa* preparations failed to produce measurable inhibition zones under the present experimental conditions. Accordingly, the null hypothesis was rejected, since combining CHX with metronidazole resulted in significantly greater antibacterial activity than CHX alone. An unexpected yet interesting observation was the absence of detectable antibacterial activity with metronidazole alone. Since *P. gingivalis* is generally considered highly susceptible to metronidazole, this finding is unlikely to reflect intrinsic resistance alone. Rather, several methodological factors may explain this observation. Agar diffusion assays depend not only on antimicrobial potency but also on the ability of the agent to diffuse through the agar matrix [21]. Metronidazole possesses different diffusion characteristics than CHX, and inadequate diffusion, incomplete dissolution, reduced stability during incubation, or insufficient local bioavailability may have limited measurable inhibition despite preserved antibacterial potential [27, 28]. Furthermore, obligate anaerobe susceptibility is influenced by inoculum density, agar composition, anaerobic conditions, and incubation methodology. Similarly, Lorenzi et al. [21] demonstrated that metronidazole exhibits dose-dependent inhibition of *P. gingivalis* and that even subinhibitory concentrations retain biological activity when appropriate experimental conditions are employed. Nevertheless, the superior antibacterial efficacy observed with the CHX–metronidazole combination is biologically plausible and consistent with previous experimental evidence [21, 29]. CHX primarily disrupts bacterial cell membranes by interacting with negatively charged phospholipids, thereby increasing membrane permeability and causing leakage of intracellular components. This membrane damage may facilitate intracellular penetration of metronidazole, which subsequently undergoes reductive activation within anaerobic bacteria and generates cytotoxic intermediates that inhibit DNA synthesis. Thus, the two agents target distinct bacterial structures, producing complementary rather than overlapping mechanisms of action. It has been reported that even concentrations of metronidazole that were ineffective when administered alone markedly potentiated the antibacterial effect of suboptimal CHX concentrations, resulting in substantial inhibition of *P. gingivalis* growth and bacterial killing [21]. The findings of the present experiment therefore further support the concept that combining membrane-active antiseptics with intracellularly acting antimicrobials may represent a promising strategy for enhancing antibacterial efficacy while potentially reducing the concentration required for each agent individually.

The present results showed that among the naturally derived products evaluated, *P. granatum* extract demonstrated considerable antibacterial activity when used alone, producing an inhibition zone that exceeded that of CHX alone. This finding agrees with previous investigations demonstrating that pomegranate-derived polyphenols, particularly punicalagin and ellagic acid, exert antibacterial activity against *P. gingivalis* through disruption of bacterial cell walls, inhibition of biofilm formation, interference with bacterial adhesion, and suppression of virulence-associated enzymes. Nevertheless, combining *P. granatum* with CHX resulted in only a modest increase in antibacterial activity compared with CHX alone and remained inferior to the CHX–metronidazole combination [15, 30, 31]. One possible explanation is that both CHX and pomegranate polyphenols predominantly target the bacterial surface and membrane, resulting in partially overlapping antimicrobial mechanisms and consequently limited pharmacological synergy. Moreover, the antibacterial activity of plant extracts depends on the concentration and stability of their bioactive constituents, which may vary according to extraction procedures and botanical composition. These factors may have limited any additional antibacterial benefit beyond that already provided by CHX. Although curcumin has been reported to possess antibacterial, antioxidant, and anti-inflammatory properties, and curcumin formulated as nanoparticles demonstrates substantially greater antibacterial activity against *P. gingivalis* [32–34], its clinical translation has consistently been hindered by poor aqueous solubility, rapid degradation under physiological conditions, and limited bioavailability [35, 36]. Therefore, the concentrations evaluated in the present study (0.1 and 1.0%) may have been insufficient to achieve effective intracellular concentrations capable of inhibiting bacterial growth.

Based upon the present experimental results, the enhanced antibacterial activity observed with CHX–metronidazole may represent a useful adjunctive approach for suppressing *P. gingivalis*, particularly in patients exhibiting elevated bacterial loads. However, host-related factors should also be considered. Habitual use of combustible and non-combustible nicotinic products adversely alters the subgingival microbiome, impairs neutrophil function, reduces tissue perfusion, and promotes persistence of periodontal pathogens, thereby potentially diminishing the clinical effectiveness of antimicrobial therapies [37, 38]. Likewise, persistent hyperglycemia, a characteristic feature of poorly controlled diabetes mellitus, promotes the accumulation of advanced glycation end-products, which may impair the host response and consequently reduce the overall effectiveness of antimicrobial therapy despite adequate suppression of the bacterial burden [9, 38, 39]. The authors applaud the study by Isola et al. [40], according to which, CHX-based oral rinses effectively reduce *P. gingivalis* carriage; however, patient-related factors, including smoking status significantly influence treatment outcomes. Future randomized clinical trials should evaluate the efficacy of CHX combined with metronidazole as an adjunct to mechanical periodontal therapy in patients with periodontitis and peri-implant diseases. Such investigations could include microbiological, clinical, and patient-reported outcome measures while stratifying participants according to smoking status, diabetic status, and glycemic control.

## Conclusion

Within the limits of the present in vitro experiment, it is concluded that the combination of CHX and metronidazole exhibits the strongest antibacterial activity, whereas *P. granatum* extracts demonstrate substantial antibacterial activity against *P. gingivalis*.
